# Lexical Disambiguation in Verb Learning: Evidence from the Conjoined-Subject Intransitive Frame in English and Mandarin Chinese

**DOI:** 10.3389/fpsyg.2016.00138

**Published:** 2016-02-16

**Authors:** Sudha Arunachalam, Kristen Syrett, YongXiang Chen

**Affiliations:** ^1^Department of Speech, Language, and Hearing Sciences, Boston UniversityBoston, MA, USA; ^2^Department of Linguistics, Rutgers University-New BrunswickNew Brunswick, NJ, USA; ^3^School of Education Science, Shanxi UniversityTaiyuan, China; ^4^Key Laboratory of Behavioral Science, Institute of Psychology, Chinese Academy of SciencesBeijing, China

**Keywords:** lexical semantics, verb learning, syntactic bootstrapping, adverbs, modification, distributivity, Mandarin Chinese, conjoined-subject intransitive

## Abstract

When presented with a novel verb in a transitive frame (*X is Ving Y*), young children typically select a causative event referent, rather than one in which agents engage in parallel, non-causative synchronous events. However, when presented with a conjoined-subject intransitive frame (*X and Y are Ving*), participants (even adults, as we show) are at chance. Although in some instances, children older than three can obtain above-chance-level performance, these experiments still appear to rely upon a within-experiment contrast with the transitive frame. This leads us to ask whether children can achieve success with the intransitive frame without such a contrast among constructions, and map a novel verb appearing in such a frame onto a non-causative meaning. Building on recent evidence that adverbial modifiers can support word learning for adjectives and for verbs (when both nominal and verbal candidate interpretations are considered) by directing children to a particular construal of a scene, we test the hypothesis that a semantically informative modifier, *together*, will provide children with additional lexical information that allows them to narrow down verb meaning and identify a non-causative interpretation for a novel verb appearing in the conjoined-subject intransitive frame. We find that for English-speaking children and adults it does, but only when *together* directly modifies the verb phrase, suggesting that participants appeal to compositionality and not just the brute addition of another word, even one that is semantically meaningful, to arrive at the intended interpretation. Children acquiring Mandarin Chinese, in contrast, do not succeed with the translation-equivalent of *together* (although adult speakers do), but they do with *dōu* (roughly, the distributive quantifier “each”). Our results point to a valuable source of information young children learning verbs: modifiers with familiar semantics.

## Introduction

Language learners the world over face the task of mapping unfamiliar words to meaning. Verbs are particularly difficult, given children's ontological expectation that new words might label objects (rather than events or properties) (e.g., Golinkoff et al., 1996; Waxman and Booth, 2001), the difficulty of identifying verb meanings simply by observing the world (e.g., Gleitman, 1990), and the relational foundations upon which verb meanings are built (e.g., Gentner, 1982). Correspondingly, experimental studies have shown that children more successfully identify novel noun meanings than novel verb meanings (e.g., Imai et al., 2008).

To overcome the challenges of acquiring verbs, children benefit from the principled relationship between the syntactic environments in which a verb appears and its semantic representation. Informative syntactic information includes the number and position of nouns (or NPs or DPs) appearing with the verb (e.g., Naigles, 1990; Fisher, 2002; Lidz et al., 2003; Gertner et al., 2006), and the range of syntactic frames in which it appears (e.g., Naigles, 1996; Gillette et al., 1999; Lee and Naigles, 2008). The *syntactic bootstrapping* hypothesis posits that observations of the syntactic environment in which a verb appears paired with knowledge of the syntax-semantics mapping can guide the child's verb learning process, and indeed syntactic bootstrapping abilities are well-established in young children (e.g., Landau and Gleitman, 1985; Naigles, 1990; Fisher et al., 1994; Fisher, 2002).

Much of the evidence documenting children's syntactic bootstrapping abilities with verbs have involved simple transitive frames, for which children map the NP/DP preceding the verb to an agentive semantic role and the NP/DP following the verb to a patient role, therefore interpreting the transitive frame as denoting a causative event. For example, in a now-classic study, Naigles (1990) presented 2-year-old English learners with a novel verb in a transitive frame (e.g., *The duck is gorping the bunny*) accompanied by a visual scene in which, simultaneously, a duck performed an action on a bunny (e.g., pushing into a bending position), and the duck and bunny each performed an action in parallel (e.g., circling their arms). At test, the events were teased apart: in one test scene, the duck performed the causative action on the bunny; in the other, both performed the synchronous (arm circling) action. Children were asked to “find gorping.” Naigles found that children who had heard the transitive frame preferred to look at the causative event over the scene with the non-causative, synchronous events. This finding has been replicated in numerous studies with children as young as 1.5 years (e.g., Naigles and Kako, 1993; Yuan and Fisher, 2009; Noble et al., 2011; Yuan et al., 2012; Arunachalam et al., 2013).

While children's understanding of the mapping between transitive frames and causative events is robust, the same cannot be said of all links between syntactic structure and semantic representations. Given that with the transitive frame, learners assign the NP/DP in the subject position an agentive role (presumably demonstrating their awareness that the preverbal argument is a subject/agent and the post-verbal argument is an object/patient), one might predict that with the conjoined-subject intransitive (e.g., *The duck and the bunny are gorping*) they would assign both of the NP/DP referents in subject position to agentive roles, and thus prefer the non-causative, synchronous events scene. However, children in these tasks do not consistently choose the synchronous scene (Naigles, 1990; Naigles and Kako, 1993; Hirsh-Pasek and Golinkoff, 1996; Arunachalam and Waxman, 2010; Noble et al., 2011).

This decalage between the transitive and intransitive frames also surfaces in the acquisition of Mandarin Chinese, interestingly so given that Mandarin (a Sino-Tibetan language) differs from English in relevant ways. For example, Mandarin verbs lack inflectional morphology indicating tense and aspect, and Mandarin allows a verb's arguments to be omitted when their referents are readily retrievable (e.g., Huang, 1984; Saito, 2007). Given the possibility of argument drop, both a transitive frame with its object dropped and an intransitive frame are realized as a subject NP or DP followed by a verb, thus rendering them indistinguishable on the surface. Mandarin learners might thus be predicted to make less use of morphosyntactic information in verb learning, as the surface properties of the sentence provide fewer cues. On the other hand, in languages that allow OV word order—possible but marked in Mandarin (Sun and Givón, 1985)—learners should have a weaker bias to map subjects to agents, and may thus find it easier to establish mappings between intransitive and non-causative referents (Chang et al., 2006). However, recent work finds that just like their English-acquiring counterparts, Mandarin-acquiring 2- and 3-year-olds take the transitive frame as a cue to causativity for both familiar verbs (Lee and Naigles, 2008) and novel verbs (Jiang and Haryu, 2014), and they are at chance in choosing between causative and synchronous scenes as referents for novel verbs in the conjoined-subject intransitive (Jiang and Haryu, 2014). Thus, at least with these two frames, Mandarin learners perform just as English learners do (see Matsuo et al., 2012 for similar results from learners of Japanese, which also permits argument drop).

This pattern raises two questions: why is the conjoined-subject intransitive frame challenging for young learners—robustly so across typologically different languages—and is there other linguistic information that might help to direct their attention to the intended interpretation? With respect to the first question, we suggest that the conjoined-subject intransitive frame is underinformative, providing insufficient semantic information for children to achieve the intended mapping given the experimental context. With respect to the second, we show that the addition of a single lexical item that highlights the semantic information that is underdetermined can support mapping to the intended, synchronous events referent.

Although we will argue that the frame was simply underinformative in the experimental contexts presented, another possibility is that children have not mastered the conjoined-subject intransitive structure (Noble et al., 2011; Gertner and Fisher, 2012), and that they instead use a strategy that does not require adult-like mapping of syntactic structure to semantic representation. For example, prior to age two, children may simply track the number and order of the nouns they hear, such that when presented with the intransitive frame, they map the first NP/DP to the Agent role and the second to the Patient role (Chang et al., 2006; Yuan and Fisher, 2009; Gertner and Fisher, 2012; Yuan et al., 2012; Messenger et al., 2014). However, at least by age two, children do not *consistently* select the causative scene as an option for both the transitive and intransitive frames, as would be predicted if they counted the nouns or consistently assigned the first NP/DP a causative agentive role in both cases. Instead, they typically perform at chance with the conjoined-subject intransitive, while consistently choosing the causative scene for the transitive frame (Arunachalam and Waxman, 2010; Noble et al., 2011). Thus, children's performance in these studies, at least for children over age two, is not likely due to systematic misinterpretation of the intransitive as a frame that signals a causative event.

Here, we adopt another perspective on the conjoined-subject intransitive, and in lieu of attributing children's performance to a lack of syntactic competence, we hypothesize that this frame provides insufficient semantic information for learners to select between the test scenes (Arunachalam and Syrett, 2014). The intransitive frame may be underinformative in at least two ways. First, it is compatible with two different construals, arising from the fact that a DP denoting a plurality occupies the subject position (e.g., Scha, 1981; Gillon, 1987; Landman, 1989a,b; Link, 1991; Verkuyl and van der Does, 1991). Under one interpretation of the conjoined subject, the *collective* interpretation, the property is predicated of the entire group (or sum) of participants, and there is one single, atomic event to which the verb refers, in which each member of the plurality is a participant. Under the second, *distributive* interpretation, the property is predicated of each event participant individually, and there are thus multiple sub-events within the larger event. Given a causative scene involving two event participants, and a non-causative scene in which two participants engage in identical, synchronous events, each scene might be a candidate for the verb in the conjoined-subject intransitive frame, but each with a different interpretation. The causative scene might be a better choice under a collective interpretation, in which both event participants, though engaged in different individual activities, may have a more general goal predicated of both. By contrast, the synchronous scene is a better choice under a distributive interpretation, because both event participants are engaged in the same lower-level action (e.g., waving), and each is therefore a participant in his/her own sub-event.

Second, a verb in a conjoined-subject intransitive frame is compatible with a range of meanings at multiple taxonomic levels. For example, it might mean something general like “playing,” “moving,” or “behaving nicely,” or it might denote a more specific action such as “waving”—which is typically the level of the intended referent in syntactic bootstrapping studies. Note that while children acquiring new nouns tend to map them at the “basic” category level (e.g., “dog”), rather than the subordinate (e.g., “beagle”), or superordinate (e.g., “animal”) level (Waxman and Markow, 1998), there is not a clear basic level for verbal event categories as there is for object categories (e.g., Huttenlocher and Lui, 1979; Maratsos and Deák, 1995). It is therefore not implausible that children might posit more general or superordinate meanings for novel verbs (Of course, this second kind of ambiguity is present for any frame, not just the conjoined-subject intransitive. A transitive frame such as, “The boy *lorped* the ball” in the context of a kicking event could be construed at multiple taxonomic levels, such as “punted,” “kicked,” or simply “touched.” Nevertheless, the primacy of causative events and the transitive-causative mapping in early childhood are well-established (e.g., Slobin, 1985; Naigles and Kako, 1993; Lidz et al., 2003; Bunger and Lidz, 2004), and so this problem is likely to be more acute for the intransitive frame).

If our claim is correct that children's difficulty with the conjoined-subject intransitive is due to the frame's underinformativity, we would predict that adults, too, would perform at chance in a task pitting causative and non-causative interpretations against each other with the intransitive frame; after all, they cannot be said to lack the requisite syntactic knowledge. For Mandarin, too, if a putative difficulty of using syntactic bootstrapping in Mandarin is not to blame for children's performance, Mandarin-speaking adults should perform at chance. We also predict that in the face of this indeterminancy, supplementing the frame with additional complementary semantic information that highlights the intended non-causative construal should increase preference for the synchronous events interpretation, for both children and adults, in both English and Mandarin. After all, even young children integrate semantic information with syntactic structure to converge on the most likely interpretation of utterances (e.g., Fisher et al., 1994). Thus, we suggest that one way in which learners might overcome the underinformativity of the intransitive frame is by attending to semantic information supplied by a co-occurring informative lexical item, which highlights the non-causative interpretation supported by the intransitive frame. Importantly, we claim that the lexical item alone is not doing all the heavy lifting: it is in virtue of its composition with the frame that it plays an important role in verb learning.

We hypothesize that adverbial modifiers may be particularly helpful in this regard, because they often carry important information about how an event unfolds, or how participants are engaged in the event. Previous research indicates that children can recruit the semantics of adverbial modifiers in adjective learning (Syrett and Lidz, 2010) and verb learning (Syrett et al., 2014). Relevant to the current goals, Syrett et al. (2014) found that the addition of the manner-of-motion adverb *slowly* to a transitive frame containing a novel verb (e.g., *Let's see a boy and a balloon. He's gonna pilk it slowly*) (a frame where children had otherwise performed at chance, because of the pronominal arguments) helped children map the verb to a meaning depicted by an appropriate motion event. Crucially, other modifiers of comparable or greater lexical frequency, or that that provided less specific information about manner of motion (*right now, nicely*) did not perform the same function (A similar pattern surfaced in Syrett and Lidz (2010) with adjectives). Syrett et al. (2014) concluded that the lexical semantics of *slowly* led children to focus their attention on the motion depicted in the event. Thus, the mere addition of another word is not enough; it must be semantically rich, sufficiently familiar to children, and directly informative about the context at hand.

Given these findings, we sought to identify an adverb that would highlight a distributive interpretation of the verb in the intransitive frame (one where each agent in subject position had the property predicated of her) and lead participants to select the scene depicting synchronous, non-causative events. We began by targeting *together*, which, under one interpretation, indicates that two events share spatial proximity and temporal contiguity (Lasersohn, 1998). Syrett and Musolino (2013, 2015) have found that 4-year-olds accept sentences with a plural subject and a VP modified by *together* (e.g., Two *boys pushed a car together*) in both collective and distributive contexts, indicating that young English-speaking children are aware of this meaning of *together*. If at age four, children allow for a distributive construal of sentences with *together*, their lexical entry for this modifier should support such a construal prior to age four, and specifically at the age range in the current study. Because plural subjects and conjoined subjects both denote a plurality, we predicted that conjoined-subject intransitive frames with *together* would support a distributive reading of the verb and direct participants to the scene in which two event participants engage in spatiotemporally coordinated (synchronous) actions. We further predicted that *together* would only support this interpretation if it served as a modifier within the verb phrase; simply hearing *together* alone, in a separate utterance, should not suffice.

Although *together* can highlight either collective or distributive interpretations, depending on the interpretations available in the context, notice that given this choice of contexts and with the addition of *together* to the intransitive frame, while the synchronous scene is an ideal instantiation of the distributive interpretation, the causative scene is not an ideal instantiation of a collective interpretation, in that it is not obviously the case that two agents are actively involved in a collective event—if there is a larger collaborative goal, it is certainly not salient. Thus, we predict that for both adults and children, *together* will highlight the distributive interpretation, leading them to view the scene with the synchronous events as the better referent.

Interestingly, our choice of adverb presents the opportunity to witness a cross-linguistic difference between English and Mandarin. To preview our results, we found that although *together* boosts performance of English-speaking children and adults, its translation-equivalent does not do so for Mandarin-speaking children—though it does for Mandarin-speaking adults. We suspected that children were simply not familiar enough with this word and its semantics to be able to benefit from it. We therefore targeted another lexical item for Mandarin learners: *dōu* is a modifier quantifier roughly equivalent to English *all, every*, or *each* (Cheng, 1995; Lin, 1998; Xiang, 2008) (although there are semantic reasons, which we will not discuss here, to think that it is not entirely equivalent to those universal quantifiers). We chose *dōu* because of independent evidence that by age 3–4 years, Mandarin preschoolers appreciate its universal quantificational import when it appears with a plural DP, and recognize that it can not only distribute a property over members of a set of individuals, but that it can be a quantificational adverb, distributing over events (Zhou and Crain, 2011), and by age four, they produce this term appropriately in their own utterances (Hsieh, 2008). We hypothesized that since preschool-age Mandarin speakers are aware of the meaning of *dōu*, just as preschool-age English speakers are aware of the meaning of *together*, and both terms invite the learner to look for a scene in which the predicate applies uniformly across event participants, both should affect interpretation of the intransitive frame and direct choices to the non-causative, synchronous scene. We found that, indeed, when presented with novel verbs in conjoined-subject intransitive frames with *dōu*, Mandarin-acquiring children also preferred the synchronous scene. Thus, it is a matter of finding the right modifier, given the language at hand.

Thus, in the current study, our goals were three-fold. First, we sought to see whether adults, like children, are at chance in choosing between causative and non-causative referents for novel verbs in the conjoined-subject intransitive, to see if child performance is more likely due to lack of syntactic competence or to the frame's lack of semantic informativity. We also aimed to replicate the previous findings with children in the same task. Second, we asked whether complementary semantic information in the form of an additional lexical item (a modifier) could help children and adults zero in on the intended (non-causative) interpretation of the verb. Finally, we asked how these aspects of verb learning play a role in English and in Mandarin, thereby highlighting both a commonality in the acquisition process across two very different languages, but also differences in the lexical information that matters.

To achieve these objectives, we used a design developed by Yuan and Fisher (2009), in which, unlike Naigles's (1990) classic paradigm, participants first hear a novel verb in (transitive or intransitive) sentences in the context of a conversation between two actors in the absence of any visual information about events. They then view two scenes side by side at test, and are asked to, “Find Ving.” To succeed, participants must have gleaned the relevant syntactic properties from the dialogues and applied that knowledge to identify which test scene best matched their syntactic/semantic representation; even before 2 years of age, children successfully map novel transitive verbs to causative events on the basis of these dialogues (e.g., Yuan et al., 2012; Arunachalam et al., 2013; Messenger et al., 2014).

Using this “dialogue” paradigm, we focused on participants' performance with the conjoined-subject intransitive, with and without an additional modifier, in English and Mandarin. For both English (Experiment 1) and Mandarin (Experiment 2), we first present the bare intransitive, no-modifier condition (Experiments 1a/2a). We then introduce a semantically informative modifier intended to direct participants' attention to the synchronous scene (Experiments 1b/2b-c). For English, we further manipulate in Experiment 1b whether the adverb modifies the VP directly or appears in a separate sentence, to determine whether the modifier enables success on its own or whether it does so only by virtue of its compositional relationship to the verb.

## Experiment 1

In Experiment 1, we begin with English-speaking adults and children. In Experiment 1a we test the hypothesis that the intransitive frame itself provides insufficient information for choosing between causative and synchronous event referents for a novel verb given our experimental design, which was modeled after many previous tasks. In Experiment 1b, we ask whether adding a relevant adverbial modifier, *together*, increases preference for the synchronous events scene, and in addition, whether it does so regardless of whether it is semantically composed with the predicate or independent of the VP.

### Experiment 1a

#### Methods

##### Participants

Participants were 15 native English-speaking adults, all students at Rutgers University—New Brunswick, and 20 children (10 males, 10 females). Two additional children were excluded from analysis for failure to provide clear pointing responses during training (see below). We targeted a relatively wide age range, from 2:4 to 3:11 (mean 3:0), because as reviewed above, previous syntactic bootstrapping work with children at about 2:3 has shown success with the transitive frame and chance performance with the conjoined-subject intransitive, and Noble et al. (2011) found that older 2-year-olds continued to perform at chance in their task, whereas 3-year-olds succeeded. We therefore included ages above and below this approximate point. Adults and older children (>3:2) were recruited from the Central NJ area, and younger children from the Boston, MA area. Populations were comparable in terms of SES. Parents signed an informed consent form approved by either Boston University or Rutgers University on behalf of their children.

##### Materials

The experiment consisted of four trials, each introducing a different novel verb. In each trial, participants were first exposed to a *Familiarization Phase*, during which they heard conversations between two actors viewed on the screen, which incorporated a novel verb eight times, always appearing in a conjoined-subject intransitive frame (see Table 1).

**Table 1:**
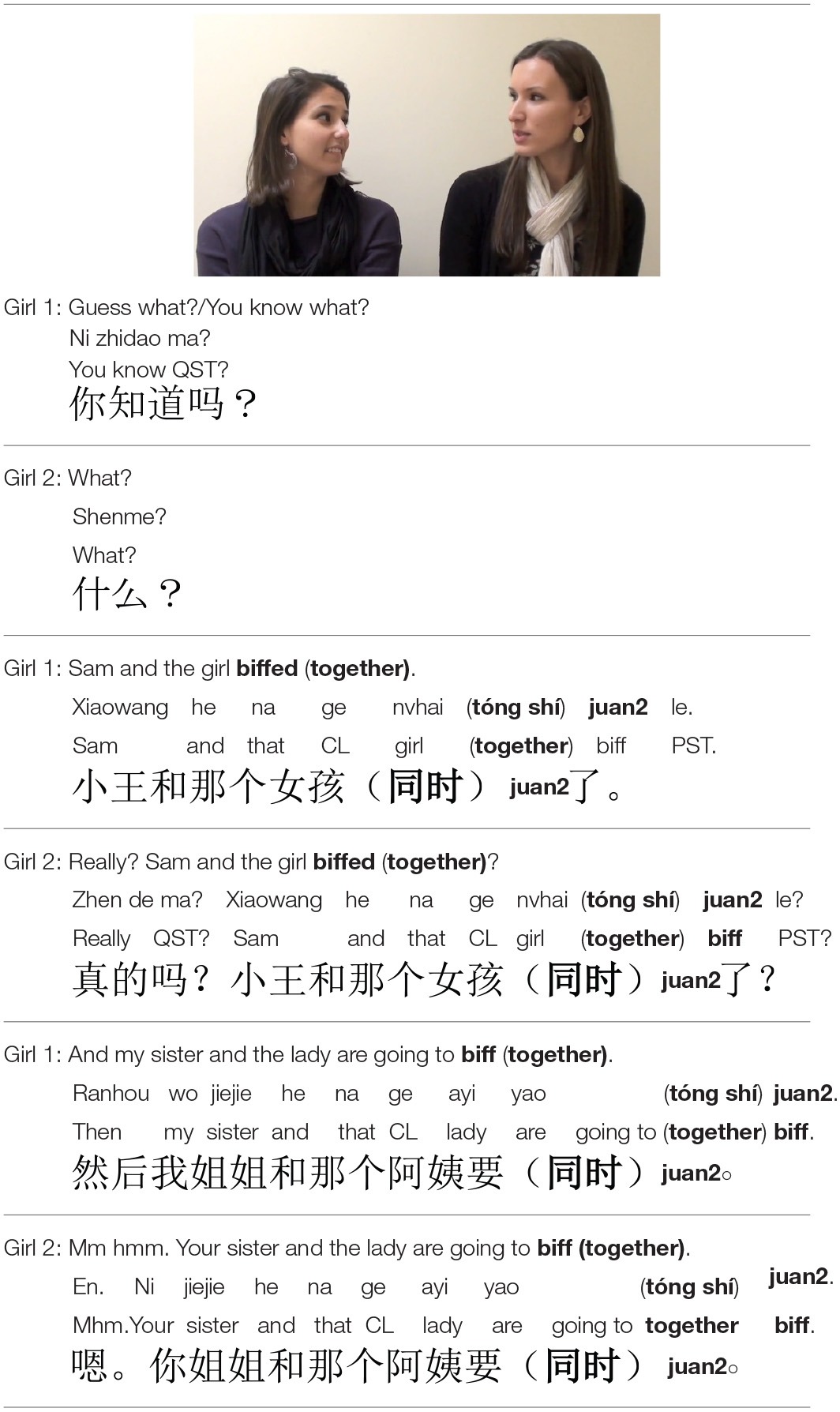
**Familiarization Phase viewed by English participants in Experiments 1a, 1b (“VP Modifier” condition) and Mandarin participants in Experiments 2a, 2b**.

Mandarin-speaking participants viewed videos of native Mandarin-speaking actors.

The *Test Phase* presented two scenes side-by-side on a white background. One was a prototypical causative scene with an agent acting on a patient, and the other a non-causative scene with actors engaged in synchronous events. The left-right position of the causative and synchronous scenes was counterbalanced across trials. Participants were directed to make a selection between the scenes (e.g., *Find biffing!*) by pointing (children) or selection on a response sheet (adults) (see Table 2).

**Table 2:**
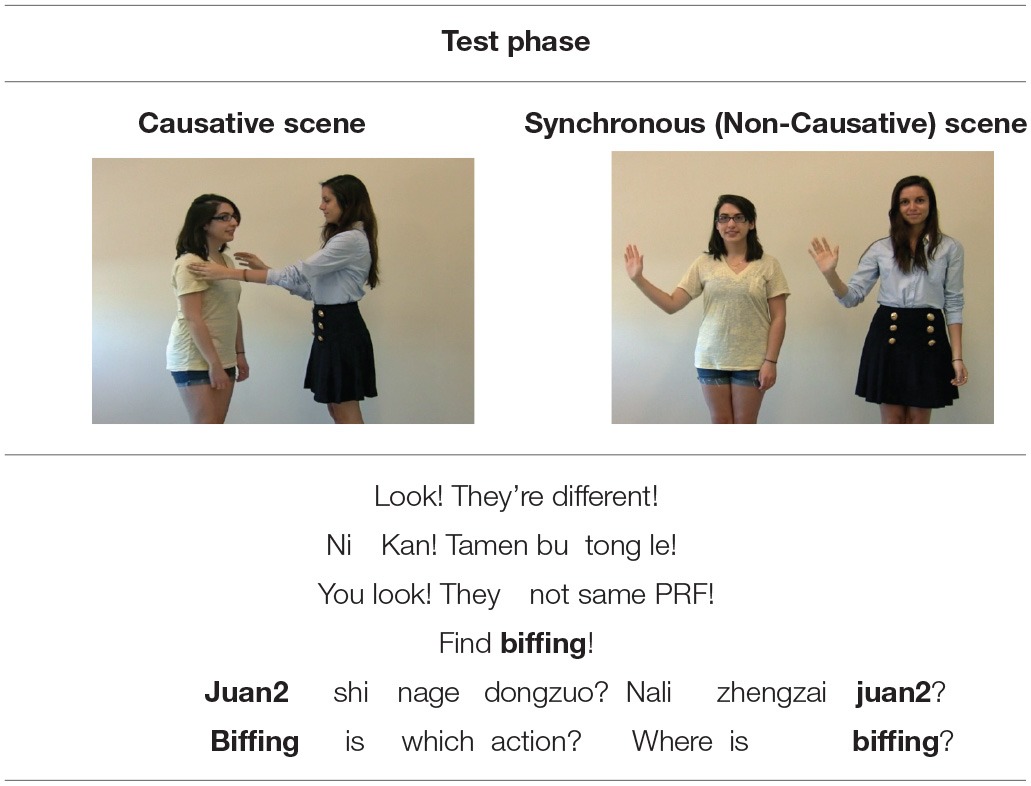
**Example of a test phase viewed by English participants in Experiments 1a, 1b (“VP Modifier” condition) and Mandarin participants in Experiments 2a, 2b**.

Mandarin-speaking participants viewed videos of Chinese actors performing these same actions.

**Figure 1:**
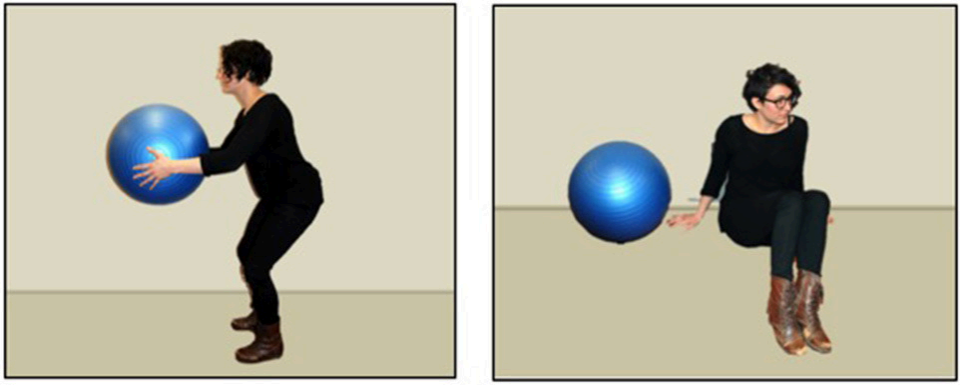
**Still images from the test scenes from one trial, depicting an animate agent (the girl) and inanimate patient (the ball)**. Mandarin-speaking participants viewed videos of Chinese actors performing these same actions.

The novel verbs were monosyllabic words faithful to the phonotactic patterns of English, and that have been used in other novel verb learning studies: *moop* (/mup/), *biff* (/bIf/), *lorp* (/l rc p/), *fez* (/fεz/). Each involved different test scenes; two trials depicted people, while two other trials involved a person and an inanimate object (e.g., a ball) see Figure 1. These test scenes were modeled closely after prior work (Arunachalam and Waxman, 2010) to maximize the interpretability of the results. We included both animate and inanimate object trials both because Arunachalam and Waxman (2010) did and because Pozzan et al. (2015) found a difference between animate and inanimate trial types. However, we found no effects of animacy in our task; thus we only report analyses with trial type collapsed.

The visual stimuli in the Familiarization and Test phases were recorded in a quiet laboratory setting. The auditory stimuli in the Test phase were recorded by a female native speaker of American English in a recording booth, using a child-directed register. Utterances were edited, controlling for intensity, using Praat (Boersma and Weenink, 2005).

##### Procedure

Participants watched the video on a computer monitor. Children indicated their choice of scene during the Test phase by pointing. Adults were given a pamphlet, with a page for each trial, and were asked to circle “LEFT” or “RIGHT.” They were not allowed to revisit pages for completed trials.

Before the experimental session proper, children participated in two training trials, designed to encourage pointing. In each, children saw two dynamic scenes side-by-side. The first depicted two familiar characters, and the second depicted two activities. The experimenter asked, e.g., “Can you find dancing?” No novel words were used during training. Those who did not point unambiguously or pointed incorrectly on both training trials were replaced in the design. On experimental trials, no feedback was provided, though child participants received general encouragement during the task.

##### Coding and analysis

Pointing responses were recorded in real time (for children), or reviewed later (for adults) by an experimenter naïve to study hypotheses. We coded which scene participants pointed to: 1 for a point to the (correct) synchronous scene, and 0 for a point to the causative scene. All participants pointed on at least three trials. Because there are two visual scenes, chance performance is 50%. We fitted the data—separately for adults and children—to a mixed-effects logistic regression model with a logit link (binomial family) with Subject and Item as random effects. We centered age around 0 by subtracting the mean age from each participant's age. Analyses were conducted using the glmer() function in R (v. 2.14.2) (Bates and Bolker, 2012; R Development Core Team, 2012). To test significance, we used the *z*-test and *p*-values output by glmer().

##### Results

*Adults*. Adults chose the synchronous scene 50% of the time. See Table 3 for means and standard deviations for all experiments. The intercept parameter estimate for the mixed-effects logistic regression model is 0.027, indicating that performance is not different from chance (probability of choosing the synchronous actions scene is 0.51). Parameter estimates for all models for all experiments are in Table 4.

**Table 3:** **Summary of participant responses (proportion of selection of Synchronous scene) across all experiments**.

		**Adult responses**		**Child responses**	
**Experiment**	**Condition**	**Mean**	**Standard deviation**	**Mean**	**Standard deviation**
**ENGLISH**					
1a	Bare intransitive	0.50	0.23	0.52	0.23
1b	*together* VP modifier	0.82	0.22	0.74	0.24
1b	*together* Predicative	0.68	0.37	0.47	0.50
**MANDARIN**					
2a	Bare intransitive	0.50	0.33	n/a	n/a
2b	*tóng shí* “together”	0.98	0.10	0.50	0.50
2c	*dōu* “each/both”	0.83	0.18	0.66	0.48

**Table 4:** **Parameter estimates from all models**.

			**Adults**			**Children**		
**Experiment**	**Condition**	**Effect**	**Estimate**	**Standard error**	***z*-value**	**Estimate**	**Standard error**	***z*-value**
**ENGLISH**								
1a	Bare intransitive	Intercept	0.024	0.50	0.048	0.089	0.45	0.20
		Age	n/a	n/a	n/a	−0.0072	0.044	−0.16
1b	*together* VP modifier	Intercept	1.69^*^	0.55	3.08	1.15^*^	0.44	2.63
		Age	n/a	n/a	n/a	0.013	0.058	0.22
1b	*together* Predicative	Intercept	1.52	0.91	1.69	−0.10	0.27	−0.38
		Age	n/a	n/a	n/a	−0.0057	0.044	−0.13
1b	*together* VP modifier vs. *together* Predicative	Intercept	1.44^*^	0.40	3.56	0.48	0.29	1.66
		Age	n/a	n/a	n/a	0.0021	0.034	0.063
		Condition (modifier vs. predicative)	0.75	0.61	1.24	1.17^*^	0.35	3.33
**MANDARIN**								
2a	Bare intransitive	Intercept	0.0054	0.36	0.015	
2b	*tóng shí* “together”	Intercept	4.81^*^	1.14	4.22	near 0	near 0	0.00
		Age	n/a	n/a	n/a	near 0	near 0	0.56
2c	*dōu* “each/both”	Intercept	1.56^*^	0.29	5.27	0.73^*^	0.30	2.40
		Age	n/a	n/a	n/a	0.13^*^	0.058	2.18
2b/2c	*dōu* “each/both” vs. *tóng shí* “together”	Intercept				0.34^*^	0.16	2.067
		Age				0.036	0.024	1.47
		Experiment (*dōu* vs. *tóng shí*)				0.68^*^	0.33	2.07

* Indicates statistical significance at or below p = 0.05.

*Children*. Like the adult participants, and like children in many other previous studies, children performed at chance. We fitted the data to a model as with the adults, but given the relatively large age range we tested, we also included Age in months as a random effect. The intercept parameter estimate for the model is 0.089 *(p* = 0.84), indicating that children's performance did not differ from chance; Age did not contribute significantly (*p* = 0.87).

##### Discussion

This experiment replicated previous verb learning studies targeting the intransitive frame by showing that (in the absence of another other within-experiment information that could favor any particular response) children do not prefer the synchronous scene as a referent for a novel verb in a conjoined-subject intransitive frame. However, in this experiment, we also added adults, who also were at chance. The results thus support our hypothesis that chance-level performance with the conjoined-subject intransitive does not stem from lack of syntactic knowledge, and that the frame itself (at the very least in this experiment and others after which it was modeled) is underinformative—a finding consistent with one pilot study with adults (Sheline et al., 2013) and a possibility entertained by Arunachalam and Waxman (2010) and Noble et al. (2011). These results lay the foundation for Experiment 1b, in which we ask whether addition of the modifier *together* increases preference for the synchronous scene.

### Experiment 1b

Experiment 1b included two conditions. Both introduced novel verbs in intransitive frames accompanied by *together*, but differing in its position. In the “VP Modifier” condition *together* appeared in the VP (e.g., *The boy and the girl biffed together*). In the “Predicative” condition *together* appeared in a separate sentence in predicative position following a copula (e.g., *The boy and the girl biffed. They were together*). This manipulation allowed us to test whether the mere presence of the modifier would support verb learning, or whether the modifier must compose with the verb to bring performance above chance level. The stimuli for the VP Modifier condition are included in Table 1. See Table 5 for the Predicative condition.

**Table 5:** **Dialogue presented to English participants during the Familiarization Phase of Experiment 1b (“Predicative” condition)**.

Girl 1: Guess what?
Girl 2: What?
Girl 1: Sam and the girl **biffed**. They were **together**.
Girl 2: Really? Sam and the girl **biffed**? They were **together**?
Girl 1: And my sister and the lady are going to **biff**. Let's see them **together**.
Girl 2: Mm hmm. Your sister and the lady are going to **biff**. Let's see them **together**.

#### Participants

Thirty native English-speaking adults, all undergraduates at Rutgers University—New Brunswick and 40 children (22 females, 18 males) (ages 2:4 to 3:11, mean 3:3) were randomly and evenly assigned to the VP Modifier or the Predicative condition. Adults and older children (>3:2) were recruited from the Central NJ area, and younger children from the Boston, MA, area. One additional adult and one child were excluded because of non-native speaker status, and seven additional children were excluded for fussiness or failure to provide clearly codable responses.

#### Materials, procedure, and analysis

These were all as in Experiment 1a, except that the linguistic stimuli during the Familiarization Phases included *together*—either as a sentence-final VP modifier in the VP Modifier condition, or in a second sentence in the Predicative condition. Participants were randomly assigned to condition in a between-subject design, which prevented participants from comparing and contrasting the linguistic material in each condition. We return to the importance of this design choice in the General Discussion.

#### Results

*Adults* chose the synchronous scene 82% of the time in the VP Modifier condition (intercept parameter *p* < 0.0021), indicating better than chance performance. In the Predicative condition, they chose the synchronous scene 68% of the time, but this was not significantly different from chance (intercept parameter *p* = 0.093). We included data from both conditions in a model with subject and item as random effects and Condition (VP Modifier vs. Predicative) as a fixed effect; we found no effect of condition (*p* = 0.22).

*Children*. chose the synchronous scene 74% of the time in the VP Modifier condition, reliably more than chance (intercept parameter *p* < 0.01), and only 47% of the time in the Predicative condition (intercept -0.10, *p* = 0.71). Neither model showed an effect of age. As with adults, we compared performance across conditions; here, however, we found an effect of condition (VP Modifier vs. Predicative) (*p* < 0.001) and no effect of age (*p* = 0.95). Adults and children thus matched our predictions that *together* would boost performance only when it composed with the predicate as a modifier, but not when it appeared elsewhere in the linguistic stimulus.

#### Discussion

In this experiment, both age groups preferred the synchronous scene if the intransitive frame was supplemented by additional semantic information, but only when it modified the VP containing the novel verb. Importantly, it cannot be the case that children's mere knowledge of the meaning of *together* as a lexical item allows them to succeed in the verb learning task; rather, the modifier must compose with the verb (or rather, the VP) to be useful.

## Experiment 2

Given the reported universality of the syntax-semantics mapping and the intransitive frame, and chance-level performance in similar previous tasks with the intransitive frame, we might expect to encounter a similar pattern to Experiment 1 in languages beyond English. In Experiment 2, we target Mandarin speakers. In Experiment 2a, we ask if Mandarin-speaking adults, like English-speaking adults, perform at chance with conjoined-subject intransitives. We test adults only, given that Jiang and Haryu (2014) have already reported that Mandarin-speaking children perform at chance through at least 4 years of age (and they report pilot data from 5-year-olds showing the same result). However, we did test 7 Mandarin-acquiring children with our experimental materials (mean age 3:8), who showed chance performance as well: 46% points to the synchronous scene). Testing adults allows us to determine, as in Experiment 1, if children's chance-level performance stems from a hypothesized difficulty due to properties of the language (i.e., argument drop), or the underinformativity of the frame. Given these results as a baseline, we then ask in Experiments 2b and 2c if other semantic information can support this mapping.

### Experiment 2a

#### Methods

##### Participants

Twenty native Mandarin-speaking adults, all undergraduate students at Shanxi University, participated. Consent was obtained according to approved procedures at Shanxi University.

##### Materials

The materials were Mandarin translations of the English stimuli used in Experiment 1a (see Table 1). A native speaker of Mandarin (the third author) translated the auditory materials into Mandarin with input from her research team and their Mandarin-English bilingual research assistants.

The dialogues and test scenes were re-recorded with native Mandarin-speaking actors in a similar manner to Experiment 1. All authors reviewed the videos to ensure that quality and length were comparable for both language groups. The four novel verbs were monosyllabic words that sounded similar to other Mandarin Chinese verbs: *juan2, mou2, fi1, cuai4*. To verify that these were novel, we presented each verb, embedded in a sentence, to 10 native Mandarin-speaking undergraduates, who confirmed that they lacked meaning.

##### Procedure, coding, and analysis

Procedure, coding, and analysis were identical to those for adult participants in Experiment 1.

##### Results

Like English-speaking adults in Experiment 1a, Mandarin-speaking adults chose the synchronous scene 50% of the time. As before, we fitted a mixed-effects logistic regression model with subject and item as random effects. The intercept parameter estimate for this model is 0.0054 (*p* = 0.99), indicating chance performance.

##### Discussion

As expected, Mandarin-speaking adults, like English-speaking adults and children, and like Mandarin-speaking children in prior work (Jiang and Haryu, 2014), were at chance. Given that the same result obtained in both English and Mandarin, for adults who have acquired the syntax-semantics mappings in their respective languages, we conclude that the conjoined-subject intransitive frame is underinformative, and that children's chance-level performance does not necessarily indicate lack of syntactic knowledge. We next asked if adding a semantically-informative modifier would boost Mandarin participants' performance as it did for English speakers. We begin with the Mandarin Chinese equivalent of *together*.

### Experiment 2b

In Experiment 2b we added a modifier, *tóng shí* “together,” whose contribution is expected to parallel that of *together* in English (We also considered a different translation of “together,” *yì qĭ*; however, native speakers told us that *tóng shí* translates roughly to “simultaneously” and more clearly highlights spatiotemporal contiguity than *yì qĭ*. Pilot testing with 27 native Mandarin-speaking adults with only one trial confirmed chance-level performance with dialogues including *yì qĭ* (14/27 synchronous scene selection), but above-chance performance with *tóng shí* (21/27 synchronous scene selection).

#### Participants

Twenty native Mandarin-speaking adults, all students at Shanxi University, and 20 children (11 females, 9 males) (ages 3:7 to 5:4; mean 4:7) participated. Note that this sample is older than the English-acquiring sample tested in Experiment 1, but importantly, Jiang and Haryu (2014) found that even 4-year-old native Mandarin speakers (and a pilot sample of 5-year-olds) performed at chance with the conjoined-subject intransitive. We therefore appealed to a large age range as we did in Experiment 1, spanning both above and below 4 years of age. Children were recruited from a kindergarten in Taiyuan, Shanxi Province, central China. As with the English-speaking children, their parents provided consent. Six additional children were excluded due to a side bias.

#### Materials

The materials were Mandarin translations of the English stimuli used in the VP Modifier condition of Experiment 1b (see Table 1).

#### Procedure and analysis

The procedure and analysis were identical to Experiment 1b (VP Modifier condition) for the English participants.

#### Results

*Adults*. Remarkably, adults now chose the synchronous scene 98% of the time. This result, in contrast to performance without *tóng shí*, indicates without a doubt that this modifier alters the mapping preference, making the scene presenting synchronous events the preferred referent. A mixed-effects logistic regression model fit to the data yielded an intercept parameter of 4.8 (*p* < 0.0001).

*Children*. Children's performance differed sharply from adults'. Overall, children selected the synchronous scene only 50% of the time. A model fit to the data as above, but including Age as a fixed effect, yielded a near-zero parameter estimate for the intercept (*p* = 1.00). Age did not contribute significantly (*p* = 0.58).

#### Discussion

Whereas, adults were pulled toward the synchronous scene, children remained at chance, like children in Jiang and Haryu (2014) and adults in Experiment 2a without the modifier. We hypothesized that these children might lack familiarity with *tóng shí*, because this term may not be sufficiently available in child-directed speech, or that the presence of another similar lexical item in the lexicon (*yì qĭ*) might result in delayed acquisition of these terms. Future research and/or corpora of child-directed speech could investigate these possibilities. For now, we ask whether some other additional lexical item can step in to boost their performance. If not, it may indicate that for Mandarin learners, integrating semantic information with the syntactic frame poses challenges.

We thus turned to another lexical item, *dōu*, which clearly predicates the target property of the individual participants in the main event, and for which we have evidence that Mandarin-speaking preschoolers are aware of certain aspects of its meaning (Hsieh, 2008; Zhou and Crain, 2011). Because *dōu* is thought to predicate of individuals to its immediate left in the syntactic structure, its presence with the conjoined subject signifies that the property expressed by the VP applies to each event participant (In this way, *dōu* is similar to the English *each*, although it is not entirely clear that this is an accurate translation or that this word is, like *each*, a quantifier. We did not attempt to test *each* with English-speaking children, because of experimental evidence that children struggle with the meaning of *each* through at least 6 years of age (see Syrett, 2015 for discussion). We predict that for adults, at least, and quite possibly also for children, *dōu* should be a clear surface cue to select the synchronous scene.

### Experiment 2c

#### Participants

Twenty adults and 20 children (10 males) (age range: 3:1 to 4:2; mean 3:10) were recruited as before. One additional adult and seven children were excluded due to a side bias.

#### Materials, procedure, and analysis

Materials, procedure, and analysis were identical to Experiment 2b, except that the target sentences contained *dōu* rather than *tóng shí* (see Table 6).

**Table 6:**
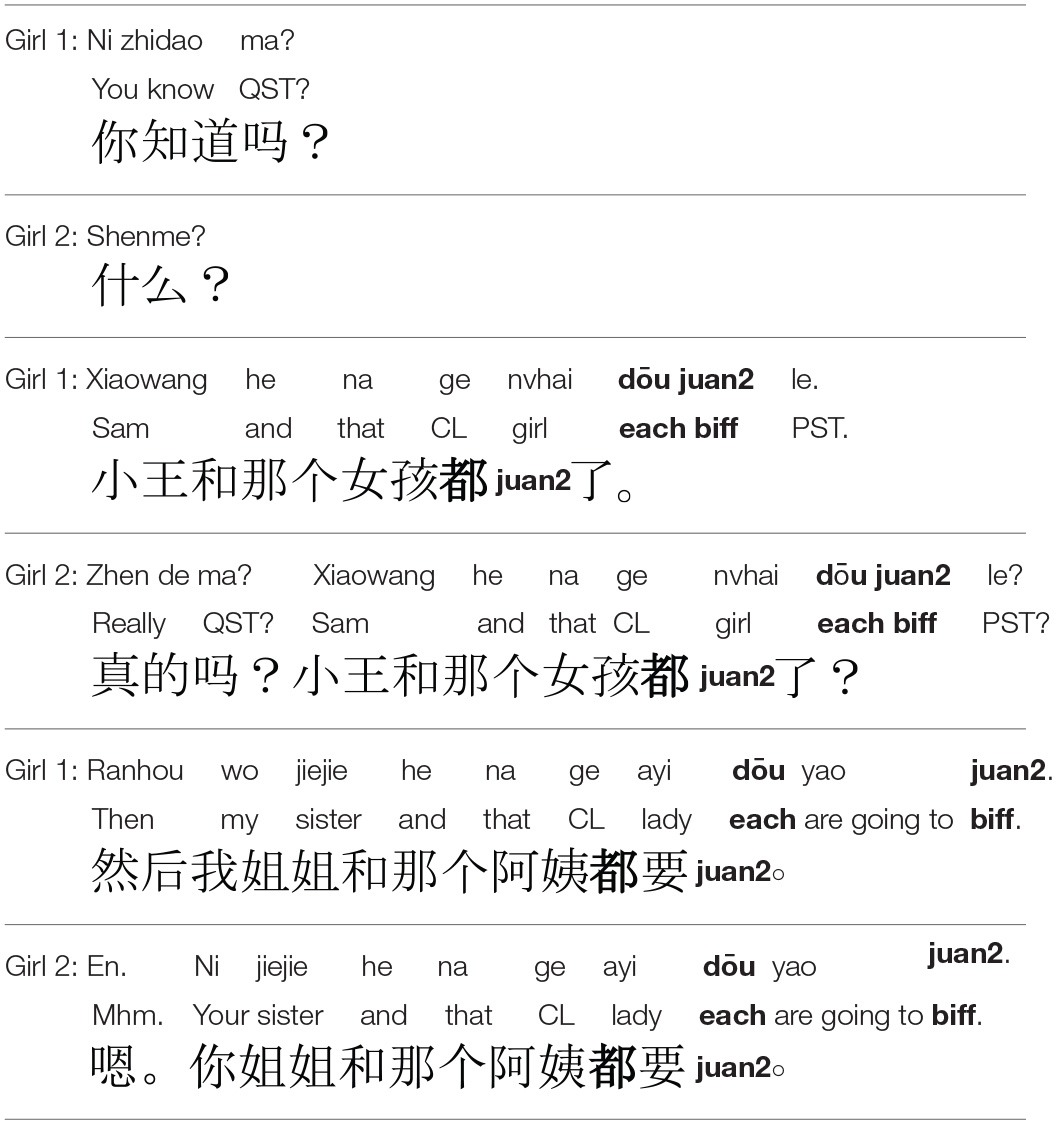
**Dialogue presented to Mandarin participants during the Familiarization Phase of Experiment 2c**.

#### Results

*Adults*. As predicted, adults chose the synchronous scene 83% of the time when presented with novel verbs in conjoined-subject intransitive frames along with *dōu*. A mixed-effects model fit to the data yielded an intercept parameter of 1.55, significant on a normal distribution (*p* < 0.001).

*Children*. This time children too were pulled above chance, exhibiting a 66% preference for the synchronous scene. As before, we fitted the data to a model including age. The intercept parameter was 0.73 (*p* < 0.02). Age was significant with a parameter estimate of 0.13 (*p* < 0.05), indicating that performance increased with age.

We further compared children's performance in Experiment 2b (*tóng shí*) with Experiment 2c (*dōu*) to see if *dōu* boosted performance significantly above performance with *tóng shí*, in addition to above chance. We fitted a model as before but including Experiment (*dōu* vs. *tóng shí*) as a fixed factor. The intercept parameter, 0.34, reached significance (*p* < 0.05), as did Experiment (0.68, *p* < 0.05), indicating that children performed significantly better with *dōu* than *tóng shí*.

These children were on average somewhat older than the English learners who succeeded with *together* in Experiment 1b, but note that they were younger than the ages at which Jiang and Haryu (2014) found failure without *dōu*. However, given that age was a significant predictor in our analysis, the ability to use this cue must increase over the preschool years. Further, research should document precisely when children become reliably able to use *dōu*, and how this relates to their acquisition of the semantics of *dōu* in other contexts.

#### Discussion

While Mandarin-speaking children remained at chance with the Mandarin equivalent of *together*, both children and adults performed above chance when the intransitive frame was supplemented with *dōu*. Our findings support prior research indicating that young Mandarin learners are familiar with *dōu* and its distributive semantics, and go further to reveal that they recruit this aspect of *dōu*'s semantic representation in the service of verb learning. That these children were younger than those in Experiment 2b but nevertheless succeeded with *dōu* supports our hypothesis that when the underspecified transitive frame is complemented by a semantically informative lexical item, this combination supports verb acquisition. Thus, Experiment 2 provides us with evidence that young Mandarin learners can recruit semantically informative modifiers to narrow the hypothesis space of meanings in the process of learning verbs, and specifically to arrive at a non-causative interpretation.

## General discussion

Our cross-linguistic investigation tested children and adults from two different language groups in the same syntactic bootstrapping task. We found that English- and Mandarin-speaking children and adults performed at chance when a novel verb occurs in a conjoined-subject intransitive frame. The findings from our child participants replicate the pattern obtained across other labs, and bolster the position that the conjoined subject intransitive frame alone does not unambiguously signal a non-causative interpretation. However, they go even further to show two things: first, that the presence of two sequential NPs in subject position does not unambiguously signal an agent-patient relationship in a causative event, at least at the ages we tested, and second, that lack of success with this frame should not be taken as a sign that participants lack the syntactic wherewithal to perform the form-meaning mapping. As adults certainly cannot be said to lack the requisite syntactic knowledge, children's inability to systematically choose between a causative and synchronous scene is not likely to reflect underdeveloped linguistic knowledge. Because the conjoined-subject intransitive frame is compatible with multiple interpretations, participants—both children and adults—who are presented with a novel verb in this syntactic frame require additional information to render a decision about the verb's intended interpretation. Thus, the syntax may signal to the learner that the novel word is a verb, but what kind of verb it is must still be resolved.

Participants are not completely at sea, though. Our tasks were designed to provide participants with additional information in the form of a semantically informative modifier. And for both adults and children, in both English and Mandarin, the presence of just one semantically informative lexical item that calls attention to the spatiotemporal contiguity of the subevents (*together* or *tóng shí*), or the distributivity of the target property over the event participants (*dōu*), swayed learners to a non-causative construal for the novel verb, pulling away from the causative scene to select the scene depicting two event participants engaged in uniform, synchronous events. Further, for English learners, the modifier was only useful when it appeared in a modifier position in the VP, strongly suggesting that our participants were integrating the semantics of *together* with the frame rather than relying on their knowledge of its lexical semantics alone. Ours are therefore the first findings to show that adverbial modification can support verb learning when two possible verbal interpretations—a causative and a non-causative one—are being entertained. Thus, like Syrett and Lidz (2010), we show that a semantically informative modifier can help the young learner assign a novel word to a more precise within-category classification.

Our findings thus highlight the importance of lexical semantics in word learning and syntactic bootstrapping. Indeed, evidence in favor of syntactic bootstrapping has often included lexical semantic knowledge as part of what children recruit to acquire verb meaning. For example, in Fisher et al.'s (1994) study, on at least some trials, children could only use the lexico-semantic content of the target words—rather than the syntactic structure itself, which was held constant—to make a selection between scenes (e.g., The bunny is fleeing the skunk/The skunk is chasing the bunny). But perhaps because the label “syntactic bootstrapping” suggests that syntactic information is paramount, most research has concentrated on the role of syntax in verb learning. The current study highlights the contribution of lexical semantics—and not just where it concerns referential information about *who* the event participants are, as in Fisher et al. (1994), but as it provides information about *how* the participants are involved in the event. As in our previous work demonstrating that the manner-of-motion adverb *slowly* supports the motion verb acquisition by providing information about the manner in which the event unfolds (Syrett et al., 2014), here we argue that the semantics of *together, tóng shí*, and *dōu*, taken together with the conjoined-subject intransitive frame, can promote one particular interpretation of the novel verb.

The ambiguity of the conjoined-subject intransitive, we have argued, results from the availability of both general and specific interpretations, as well as the ambiguity inherent in the plural subject. We suspect that in many of the early syntactic bootstrapping studies, the assumption behind the choice of the conjoined-subject intransitive as a control for the transitive was the hypothesis that both event participants named in the subject would be perceived as agentive and thus mapped onto agentive roles, which is depicted most clearly in the synchronous scene, and that the absence of a direct object would signal lack of causativity (see discussion in Naigles and Kako, 1993) Indeed, Dowty (1991) raises this assumption in his discussion of proto-role alignment in language acquisition: “When confronted with a predicate denoting a kind of event that can reasonably be understood as either symmetrically or asymmetrically volitional (or motional), does the learner automatically assume that the collective-subject version [ = conjoined-subject intransitive] is symmetrically volitional (or motional) and the two-place version asymmetrically volitional (or motional), without requiring any specific empirical data to that effect? If so, then the proto-roles and their alignment principle would be functioning as a kind of “semantic default” for the learning of lexical meaning” (p. 586).

Much research has shown that something like Dowty's (1991) proto-role alignment does indeed guide early verb learning, with children initially assigning agentive roles to the referents of NPs/DPs in subject position and patient roles to the referents of DPs in object position of a transitive verb (e.g., Gertner et al., 2006). However, our current findings illustrate that adults' and children's understanding of proto-agentiveness is not monolithic, but rather differs across contexts. This is precisely why we chose to test the same scene types used in previous syntactic bootstrapping studies (which are, after all, merely candidates among the alternative interpretations that children must be able to assign to utterances). Our research thus uncovers a nuance with respect to syntactic bootstrapping with the conjoined-subject intransitive: these agents can either coordinate their involvement in a single event (allowing the predicate to apply to the group of agents collectively), or act individually in separate, but coordinated, events (allowing the predicate to be distributed over event participants), and these two possibilities lead to different interpretations.

Strikingly similar across our experiments was English and Mandarin speakers' chance performance with novel verbs in a bare conjoined-subject intransitive frame: adults and children from both language groups showed no preference for the synchronous scene. Also similar is participants' ability to use an additional, semantically informative lexical item to focus attention on the synchronous scene. Because Mandarin and English differ in many respects relevant for syntactic bootstrapping, such as the availability of argument drop and the inflectional morphology on verbs, this pattern strongly hints at universal expectations or biases underlying verb acquisition (whatever the source of these expectations may be). This is particularly interesting given the claim in the literature that Mandarin is a “verb friendly” language compared to English (see, e.g., Bornstein et al., 2004; Waxman et al., 2013, for reviews). Experimental verb learning work has not thus far supported this hypothesis (Imai et al., 2008; Leddon et al., 2011). In fact, we initially hypothesized that Mandarin learners might struggle in this type of verb learning task, because of the scarcity of verbal inflectional morphology and the prevalence of argument drop. Nevertheless, our results show that Mandarin learners perform strikingly similarly to English learners. The only difference we found between the two languages had to do with the particular lexical item that boosts performance. This contrast suggests that not just any lexical item—semantically informative or not—will do: it needs to be one that has both the requisite lexical semantic properties that support the mapping *and* that is sufficiently familiar to children to allow them to access its semantics.

Although our findings of chance performance with the conjoined-subject intransitive when no modifier is present are consistent with much of the previous literature as reviewed above (Naigles and Kako, 1993; Hirsh-Pasek and Golinkoff, 1996; Arunachalam and Waxman, 2010; Noble et al., 2011), it is also the case that some studies have shown different results. For example, Noble et al. (2011) and Noble et al. (2016) reported that children in their older age groups (no younger than 3:4) *did* preferentially select the synchronous scene over the causative one, and Pozzan et al. (2015) found the same for children from age 3:1 to 4:8, at least in trials with animate agents and inanimate theme participants, and for adults. Recall however that in the current study we found chance performance in the relatively large age group we sampled as well as with adults. What could account for the discrepancy between our results and theirs?

We suspect that the differences are primarily methodological. In our experiments, participants were only presented with the linguistic information prior to the visual stimuli, while Noble et al. (2011), Noble et al. (2016), and Pozzan et al. (2015) also presented the linguistic information simultaneously with the visual scenes. When participants encounter the syntax first, they have to posit a representation for the verb in the absence of any candidate referents. If the frame is underinformative, participants may begin with an underspecified representation, and may struggle to identify the best scene choice at test. By contrast, if older child participants or adults are presented with the visual information at least once simultaneously with the linguistic information, they may be driven to make their choice of scene based on a more strategic guess as to which scene best matches all the information they are given. As we have noted, children often hear verbs in the absence of the events they describe (Tomasello and Kruger, 1992), and so it is important to understand the representations learners may begin with when hearing linguistic information alone.

Relatedly, another key difference between the studies by Noble et al. (2011), Noble et al. (2016), and Pozzan et al. (2015) on one hand and our study (and the others reviewed above) on the other is that these two studies in particular employed a within-subject design, such that the same participant heard both transitive and intransitive sentences within an experimental session. It is possible that a within-subject design leads children to expect that they *should* map verbs appearing in a transitive frame to a causative event, as they would normally do, but that a verb appearing in the (other) conjoined-subject intransitive frame *should not* be paired to a causative event. Given this design, children might employ something like the Principle of Contrast (Clark, 1987) to infer that the intransitive frame—which is compatible with either scene and interpretation—should be interpreted as a cue to a non-causal interpretation. Thus, it is entirely possible that in our study children and adults performed at chance with the conjoined-subject intransitive because while they possess the requisite knowledge of the syntax and syntax-semantics mapping, the frame is not informative enough on its own.

Gertner and Fisher (2012) and Noble et al. (2016) also observed successful mapping of conjoined-subject intransitives to synchronous scenes when the (distractor) test scene depicting the causative event depicted the second-named event participant as the agent, instead of the patient (e.g., for *The boy and the girl biffed*, the causative test scene depicts the girl acting on the boy). In this case, another cue contributes to the synchronous scenes interpretation—that the first participant mentioned is *not* a causal agent in the causative scene.

While these differences are interesting and merit future study, they do not undermine the contribution of our current research, which is that, in a design in which the conjoined-subject intransitive frame provides insufficient information for disambiguation, and learners consistently are at chance between causative and synchronous interpretations, a modifier with the appropriate semantics can step in to steer them toward a synchronous interpretation. Thus, we have uncovered an additional source of information that supports verb learning in both English-speaking and Mandarin-speaking children.

The context-specific nature of the finding underscores a point we raised early on in discussing the semantics of *together*: The contribution of a modifier to learners' interpretation of the conjoined-subject intransitive is context-dependent; the most appropriate referent depends to some extent on the available candidate scenes. This is not to say that the modifier itself has a context-dependent semantics, but that the meaning highlighted by a modifier will depend on the context in which it occurs. In our task, a causative scene was pitted against a synchronous scene, making the latter a better target, since it aptly highlighted the meaning of *together* in which there are spatiotemporally contiguous events. However, if a causative scene were instead pitted against one for which neither a collective nor a distributive interpretation were salient, then we might predict that the causative scene could be the better referent, because *one* might, depending on the activity, access a collective construal. Relatedly, we suspect adults and children only succeeded when *together* appeared in the verb phrase as a modifier rather than in a separate utterance because the modifier must compose with the predicate to yield the intended interpretation.

We therefore wish to emphasize two points. First, the meaning highlighted by a potentially polysemous lexical item (such as *together*) is subject to the events portrayed in the scene(s) at hand. Second, this lexical item can alter the learner's construal of the scene, highlighting whatever aspect is most relevant given the context. This is in fact an interesting difference between the role that modifiers such as *together* can play in syntactic bootstrapping and the role played by more commonly studied elements, such as the referential information available in contentful phrases such as *the bunny*; while the possible referents of *the bunny* remain relatively invariant across contexts (that is, referring to a member of a particular species, unless used figuratively), modifiers must compose with the predicate to derive their meaning, and have varying interpretations depending on both the semantics of the predicate and the context.

Taken together, our findings make several contributions to the study of verb learning. First, they provide a plausible explanation for the chance-level performance with the conjoined-subject intransitive evident in verb learning experiments for decades: the frame is underinformative, allowing for a meaning supported by either context. Second, they demonstrate that in cases where syntactic structure alone is insufficient to adjudicate between possible interpretations of a novel verb, learners can take advantage of the lexical semantic information contributed by non-argument elements that specify how participants are engaged in the event(s), and narrow down the meaning of the verb. Finally, our results reveal that providing additional lexical semantic information helps Mandarin and English learners alike. While the particulars of *what kind* of additional information supports learning differ, the overall *strategy* of integrating syntactic and semantic information to arrive at a novel verb's meaning is shared.

## Author contributions

All authors listed, have made substantial, direct and intellectual contribution to the work, and approved it for publication.

## Funding

This work was made possible by NIH grant K01DC013306 to the first author, and funding provided to the second author in the form of a Rutgers Startup grant, the Aresty Research Center at Rutgers-New Brunswick, and a collaborative grant for a research exchange between the Rutgers Center for Cognitive Science (RuCCS) at Rutgers, The State University of New Jersey—New Brunswick and the Institute of Psychology of the Chinese Academy of Sciences (KJZD-EW-L04), Beijing, sponsored by the Rutgers School of Arts and Sciences and the Chinese Institute of Psychology.

### Conflict of interest statement

The authors declare that the research was conducted in the absence of any commercial or financial relationships that could be construed as a potential conflict of interest.
